# Recombinant Domain of Flagellin Promotes In Vitro a Chemotactic Inflammatory Profile in Human Immune Cells Independently of a Dendritic Cell Phenotype

**DOI:** 10.3390/molecules28052394

**Published:** 2023-03-05

**Authors:** Roxana González-Stegmaier, Adam Aguirre, Constanza Cárcamo, Patricia Aguila-Torres, Franz Villarroel-Espíndola

**Affiliations:** 1 Translational Medicine Laboratory, Instituto Oncológico Fundación Arturo López Pérez, Santiago 8320000, Chile; 2 Laboratorio de Microbiología Molecular, Escuela de Tecnología Médica, Universidad Austral de Chile, Puerto Montt 5480000, Chile

**Keywords:** flagellin, TLR5, PAMPs, cytokines, immunomodulation

## Abstract

Flagellin is the major component of the flagellum in gram-positive and -negative bacteria and is also the ligand for the Toll-like receptor 5 (TLR5). The activation of TLR5 promotes the expression of proinflammatory cytokines and chemokines and the subsequent activation of T cells. This study evaluated a recombinant domain from the amino-terminus D1 domain (rND1) of flagellin from *Vibrio anguillarum*, a fish pathogen, as an immunomodulator in human peripheral blood mononuclear cells (PBMCs) and monocyte-derived dendritic cells (MoDCs). We demonstrated that rND1 induced an upregulation of proinflammatory cytokines in PBMCs, characterized at the transcriptional level by an expression peak of 220-fold for IL-1β, 20-fold for IL-8, and 65-fold for TNF-α. In addition, at the protein level, 29 cytokines and chemokines were evaluated in the supernatant and were correlated with a chemotactic signature. MoDCs treated with rND1 showed low levels of co-stimulatory and HLA-DR molecules and kept an immature phenotype with a decreased phagocytosis of dextran. We probed that rND1 from a non-human pathogen promotes modulation in human cells, and it may be considered for further studies in adjuvant therapies based on pathogen-associated patterns (PAMPs).

## 1. Introduction

The innate immune system plays an essential role in the host defense against infections by sensing pathogens and directing adaptative immune responses to the infections [1]. The recognition of pathogen-associated molecular patterns (PAMPs) by Toll-like receptors (TLRs) allows for an innate immune response, triggering the expression of proinflammatory cytokines and the induction of co-stimulatory molecules on antigen-presenting cells (APCs) [2,3]. Flagellin is the main protein component of flagellum in gram-positive and -negative bacteria and the ligand for the TLR5 in several organisms, and its binding activates a broad range of cell types within the innate and adaptive immune system to promote cytokine production [4,5]. TLR5 is expressed constitutively in immune cells, such as monocytes and immature DCs, as well as in epithelial cells [6]. Due to their ability to connect the innate with the adaptative immune response, TLR agonists are promising as adjuvants in vaccines against infectious diseases and complex diseases, such as cancer [7,8]. The adjuvant activity of flagellin has been reported in several experimental vaccines, with a broad diversity of antigens derived from the microorganisms responsible for several infectious diseases, such as influenza [9,10], West Nile fever [11], malaria [12], plague [13], and tuberculosis [14]. In addition, some antitumor vaccines that use flagellin as an adjuvant have shown to be successful in clinical trials [8,15]. In a mice model, the combined administration of *Vibrio vulnificus* Flagellin B and Human papillomavirus (HPV) E6/E7-derived peptides has shown an effective anti-tumor response by T-cells activation, which seemed to be independent of the TLR5 expression on the tumor cell surface [16,17]. In a melanoma model, a combined regimen of vaccination, based on a flagellin-adjuvanted tumor-specific peptide and photodynamic therapy, showed a systemic and local response of peptide tumor antigen-specific IFNγ-secretion and accumulation of effector memory CD8+ T cells [18], and that immune response was enhanced when an immune checkpoint inhibitor was used in parallel. Similar results were observed in a glioma model, where a prophylactic subcutaneous administration with glioma whole extract and flagellin induced a potent cytotoxic activity and prolonged the survival of GL261-bearing mice; however, that regimen was not therapeutically efficient once the tumor was established [19].

Flagellin’s potential has been explored in oncology and demonstrated the capacity to inhibit the tumor growth and tissue damage associated with radiation [20,21,22,23]. In fact, the treatments with entolimod, a Food and Drug Administration (FDA) approved drug, reduced radiation-induced apoptosis and accelerated the regeneration of progenitors in radiation-damaged tissues [24].

Previously, we demonstrated that a recombinant including the Ala45–Tyr176 region from the amino terminus D1 domain of flagellin (rND1) from *Vibrio anguillarum* has a strong immunomodulatory role in lower vertebrates in vitro and in vivo [25,26], and considering the structural similarities of the flagellum in fish and human pathogens, we have hypothesized that rND1 may activate human TLR5, as well. Therefore, this study aims to evaluate, in vitro, the response of human peripheral blood mononuclear cells (PBMCs) and monocyte-derived dendritic cells (MoDCs) exposed to rND1. As expected, the results showed an induction of pro-inflammatory cytokines, such as IL-1β, TNF-α, and IL-8, at transcriptional level, as well as a profile of secreted proteins concordant with a chemotactic signature in mononuclear cells. This contribution is the first step for considering the use of rND1 from a non-human pathogen to promote immune modulation in human cells, and it could be considered for further studies in adjuvant therapies based on the activation of the immune response by PAMPs.

## 2. Results

### 2.1. rND1 Is Not Toxic and Promotes an In Vitro Proinflammatory Response in Human Immune Cells

**Figure 1:**
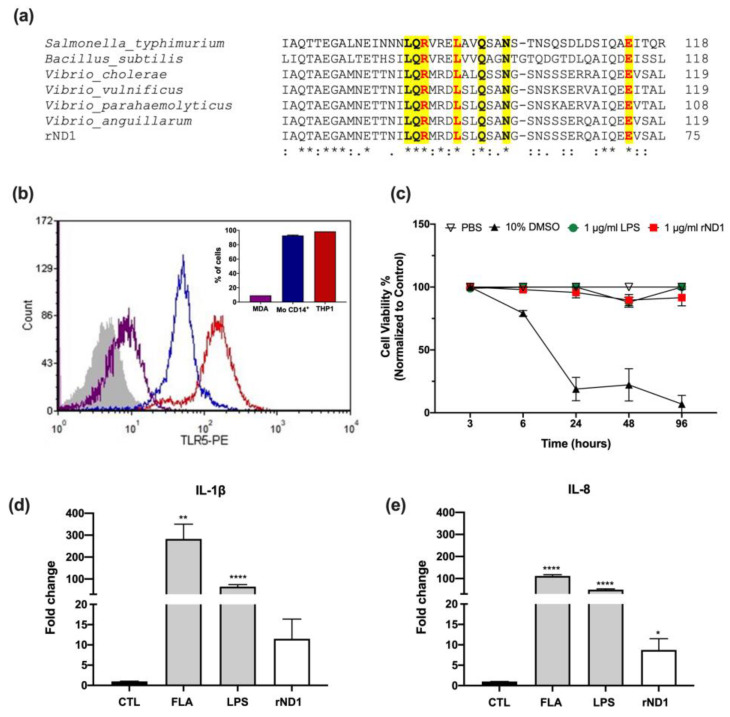
rND1 promotes an in vitro proinflammatory response in THP-1 cells. (**a**) Multiple alignment of the flagellin amino acid sequences from human and fish pathogens, conserved amino acid residues in rND1 are marked in bold. They key residues required for TLR5 are highlighted in yellow [5] and in red [27]; (**b**) Analysis of the expression of TLR5 by flow cytometry, histogram represents the TLR5 detection in monocytes (Mo CD14+, blue), THP-1 cells (THP1, red), MDA-MB-231 breast cancer cells (MDA, purple), and a control without primary antibody (gray). The bar graph shows the percentage of positive cells for each analyzed type; (**c**) Effect of rND1 on cell viability in THP-1 cells. The data is presented as mean ± SEM for three independent experiments and normalized to vehicle control (PBS); (**d**) IL-1β and (**e**) IL-8 in THP-1 cell line stimulated with rND1 1μg/mL for 3 h. Values of fold change were calculated from the unstimulated control (CTL) after being normalized to the expression of GAPDH and β-actin. Results are presented as the mean ± SEM in triplicate. Asterisks indicate significant differences, compared to the unstimulated control and analyzed with student t-test. FLA: Flagellin from *S. typhimurium* (50 ng/mL); LPS: Lipopolysaccharide from *E. coli* K12 strain (1 μg/mL). * *p* < 0.05; ** *p* < 0.01, *** *p* < 0.001, and **** *p* < 0.0001.

Using in silico multiple sequence alignment, we compared the similarities between the amino acid sequences of the domain D1 in different flagellin proteins, from both human and fish pathogens, and rND1 showed a 92% of identity with *Vibrio cholera*, 83% with *Vibrio vulnificus*, 85% with *Vibrio parahaemolyticus*, 42% with *Salmonella typhimurium*, and 43% with *Bacillus subtilis*. Based on bioinformatics analysis, we verified that rND1 contains key conserved amino acids residues, which are relevant for the activation of TLR5 in higher mammals (Figure 1a), and we have also estimated that the dose previously used in teleost fish (*Sparus aurata* L., Sparidae and *Oncorhynchus mykiss*, Salmonidae) will induce a comparable cytokine effect in human monocytes [25].

Initially, our results confirmed the expression of TLR5 in a human monocyte line (THP-1) and isolated human CD14+ monocytes (Mo) using flow cytometry (Figure 1b,c); in addition, the MDA-MB-231 breast cancer line was used as non-immune cell control. Using 3-[4,5-dimethylthiazol-2-yl]-2,5 diphenyl tetrazolium bromide (MTT) assay, we proved that rND1 does not induce a deleterious effect on human cells at the dose of 1 μg/mL (Figure 1d). No changes in THP-1 cell viability were observed during the first 24 h; however, a longer incubation (up to 72 h) showed a reduction on MTT metabolization by 10%, but it was not statistically significant (*p*-value = 0.7313) (Figure 1d).

To confirm that the recombinant domain is biologically active in human cells, THP-1 cells were stimulated for 3 h with 1 μg/mL of rND1, showing a significant upregulation of IL-1β (11.4-fold) and IL-8 (8.7-fold), compared to the unstimulated control (Figure 1d,e). In parallel, THP-1 cells were stimulated with a commercial of two PAMPs as internal controls, including lipopolysaccharide from *E. coli* K12 strain (LPS) and flagellin from *S. typhimurium* (FLA), and it showed, for IL-1β, an upregulation by 65-fold and 282-fold; for IL-8, the transcript increased by 49-fold and 111-fold, respectively (Figure 1d,e).

**Figure 2:**
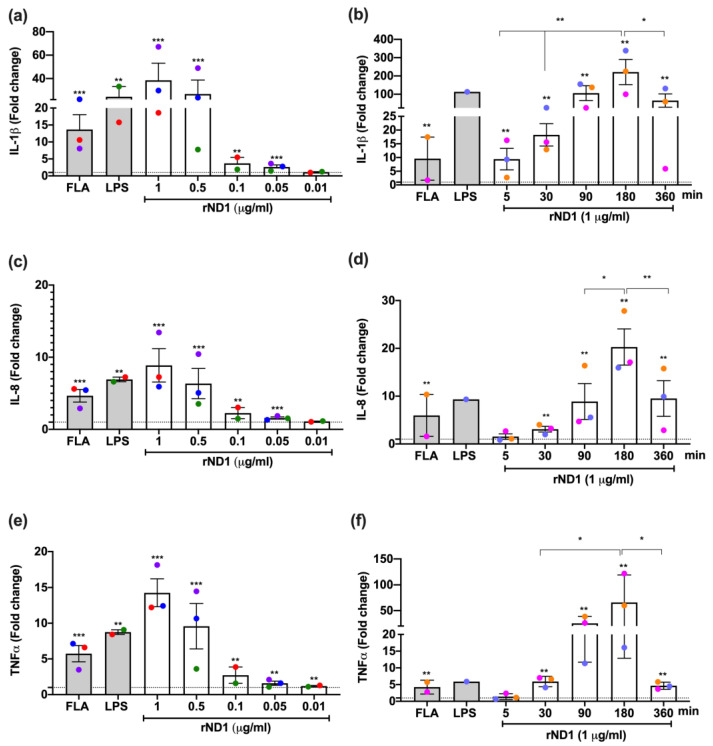
rND1 promotes a dose- and time-dependent in vitro pro-inflammatory response in mononuclear cells. Values of fold change were calculated from the unstimulated control after being normalized to the expression of GAPDH and β-actin; (**a**,**c**,**e**) Dose response and (**b**,**d**,**f**) Time response of mononuclear cells stimulated with rND1 for 3 h. The expression levels for IL-1β, IL-8, TNF-α are presented as the mean ± SEM, and statistical differences from the Mann–Whitney test are shown. FLA: Flagellin from *S. typhimurium* (50 ng/mL); LPS: Lipopolysaccharide from *E. coli* k12 strain (1 μg/mL). Color circles correspond to each event of monocytes isolation from each of the healthy volunteers in independent experiments. In all cases, the dashed line corresponds to the unstimulated control. * *p* < 0.05; ** *p* < 0.01 and *** *p* < 0.001.

The rND1 and TLR5 interaction in non-transformed cells were primary measured as the cytokine mRNA induction in mononuclear cells (Mo) isolated from healthy donors and stimulated in vitro with different concentrations of rND1 for 3 h. The results showed an expression of proinflammatory cytokines in a concentration dependent manner, with a response peak between 0.5 and 1 μg/mL of rND1 (Figure 2a,c,e). As we observed before in teleost, the maximum expression peak was at 1 μg/mL, with a mean upregulation for IL-1β above 30-fold, for IL-8 by 8-fold, and for TNF-α by 14-fold (Figure 2a,c,e).

Once we confirmed that rND1 increases the expression of proinflammatory cytokines in Mo cells in a dose-dependent manner, a similar experiment was performed at different time of incubation. The results showed a gradual upregulation of cytokines up to 360 min after stimulation. We observed a maximum expression peak at 3 h (180 min), with a statistically significant increase of 220-fold for IL-1β (Figure 2b), 20-fold for IL-8 (Figure 2d), and 65-fold for TNF-α (Figure 2f), compared to the unstimulated control. More than 3 h of stimulation did not represent a significant upregulation, and in some cases, a reduction on the cytokine mRNA levels was observed at 6 h.

Even though we observed a difference in the magnitude of the cytokine upregulation between experiments, it was not related to the cell isolation and culture, suggesting a host factor was not considered during the blood donor selection (Supplementary Material Table S1). Overall, rND1 showed a differential induction capacity on THP-1 and Mo cells. To avoid discrepancies between cell types and TLR’s functionality, FLA and LPS were included in each assay as functional controls; however, both THP-1 and Mo differentially responded to FLA and LPS (Figure 1 and Figure 2), mainly for IL-8 induction.

### 2.2. rND1 Promotes an In Vitro Chemotactic Response in Human Immune Cells

**Figure 3:**
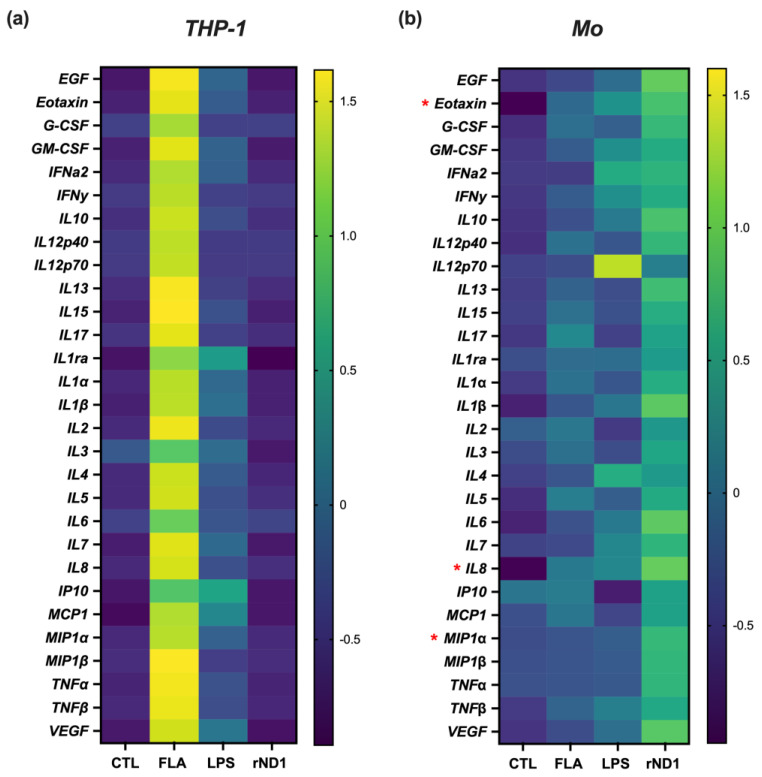
THP-1 cells and mononuclear cells (Mo) showed a differential cytokines profile after stimulation with rND1. The secreted pattern of cytokine in both (**a**) THP-1 and (**b**) Mo cells after 3 h of incubation with 1 µg/mL rND1 is shown in a heat map. Values of each cytokine were normalized to z-score. Each cytokine is presented as the mean of several independent experiments in THP1 (n = 3) and Mo (n = 4). FLA: Flagellin from *S. typhimurium* (50 ng/mL); LPS: Lipopolysaccharide from *E. coli* k12 strain (1 μg/mL). Red Asterisks indicate significant differences between rND1, compared to the unstimulated control by One-way ANOVA and Tukey’s multiple comparison tests. Cytokine values in pg/mL are shown in Tables S1 and S2 of Supplementary Materials. * *p* < 0.05.

Knowing the optimal condition for rND1 to induce a proinflammatory status, 29 cytokines and chemokines secreted to the culture medium were quantified, and the induced profile in THP-1 and Mo cells was compared as a heat map (Figure 3). Both, FLA and LPS induced the secretion of multiple cytokines in THP-1 cells with very distinctive patterns; however, rND1 did not show a significant cytokine production (Figure 3a, Supplementary Materials Table S2). These results are consistent with those obtained for the IL-1β and IL-8 transcripts, where THP-1 cells responded mainly to FLA and LPS and slightly to rND1 (Figure 1d,e). Opposite to the monocyte-derived cell line (THP-1), the Mo cells stimulated with rND1 showed a strong response with a very characteristic pattern (Figure 3b, Supplementary Materials Table S3). This cytokine’s pool was concordant with a chemotactic profile, which was significant, compared to the control without treatment. The most significantly released molecules were eotaxin (*p*-value = 0.038), MIP1α (*p*-value = 0.046), and IL-8 (*p*-value = 0.037). As we observed before, full FLA and LPS showed lower activity than rND1, even at the protein and at the mRNA levels (Figure 2c,d and Figure 3b).

### 2.3. Monocyte- Derived Dendritic Cell (MoDC) Are Insensitive to rND1 Stimulation

**Figure 4:**
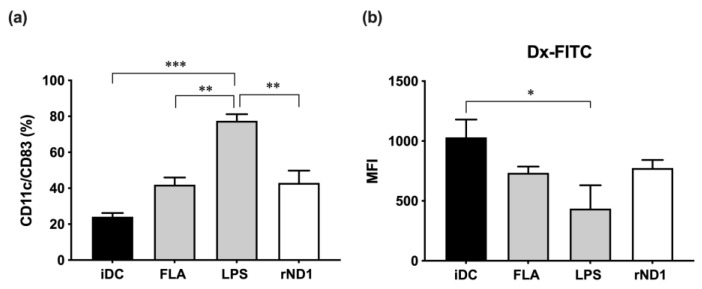
Monocyte-derived dendritic cells are insensitive to rND1 stimulation. (**a**) Percentage of expression of CD83 in CD11c + DC in iDC, and FLA, LPS, and rND1 treated cells for 18 h. The values represent the means of three independent experiments; (**b**) Median fluorescent intensity associated with the uptake of FITC-dextran by iDC, treated with FLA, LPS, and rND1 for 18 h, respectively. One-way ANOVA and Tukey’s multiple comparison tests. iDC: immadure dendritic cell; mDC: mature dendritic cell. FLA: Flagellin from *S. typhimurium* (100 ng/mL); LPS: Lipopolysaccharide from *E. coli* K12 strain (1 μg/mL); rND1: recombinant amino terminus D1 domain from *V. anguillarum* flagellin (5 μg/mL). * *p* < 0.05; ** *p* < 0.01, and *** *p* < 0.001.

The adjuvant role of flagellin and derived peptides may influence dendritic cell functions; for this purpose, CD14+ monocytes were isolated from unrelated healthy volunteers and cultured in presence of IL-4 and GM-CSF to induce differentiation to dendritic cells. After 6 days of culture, cells were independently stimulated with rND1 5 μg/mL, FLA 100 ng/mL, LPS 1 μg/mL, and untreated control (vehicle) for 18 h, and CD83 was measured in mature MoDC. The results showed that the treatment of iDC with rND1 or FLA slightly induced an increase of CD83 positivity, suggesting an intermediate DC profile, which was significantly lower, compared to LPS (Figure 4a, Supplementary Materials Figure S1). The observed phenotype in MoDC stimulated with LPS was considered as the maximum level of maturity (Figure 4a).

Interestingly, when rND1 was evaluated, the phagocytic capacity was lower than the iDC, and the same pattern was observed for the full FLA (Figure 4b). In addition, other phenotypic changes were studied, such as the presence of co-stimulatory molecules and HLA class II molecules on the cell surface. When molecules such as CD86, CD40, and HLA-DR were measured in treated cells, none of them showed a significant upregulation after incubation with rND1, FLA, or LPS; however, there is a clear trend for LPS to upregulate those molecules (Supplementary Materials, Figure S2). Together, these results suggest that monocyte-derived dendritic cells treated with rND1 at high doses and long-term incubation have a transition phenotype between immature and mature dendritic cells, characterized by a decreased phagocytic capacity and low expression of costimulatory molecules.

## 3. Discussion

In this work, we explored the capacity of the recombinant amino terminus D1 domain (rND1) of flagellin B from *V. anguillarum* to induce, in vitro, an effective proinflammatory status in human mononuclear cells. This recombinant domain (rND1) contains key amino acids needed to bind TLR5, and it has shown IL-1β, TNF-α, and IL-8 overexpression in mammals and non-mammalian models [25,26,28,29,30]. Specifically, truncated flagellin from *Salmonella dublin* containing only the conserved N and C domains (ND1/2ECHCD2/1) stimulated the secretion of IL-8 on Caco-2BBe and T-84 cells in a similar level to the full-flagellin, but when a truncated flagellin version was used, containing only the D3 domain, IL-8 secretion was not observed [29]. Similar results were obtained from THP-1 cells stimulated with truncated flagellins form *Treponema pallidum*; the authors considered truncated version D1 domain in N-terminus, C-terminus, and N and C terminus together, and these recombinants failed to induce IL-6 and IL-8 upregulation, and only the truncated flagellin in hypervariable segment generated IL-6 and IL-8 expression with comparable results to the wildtype flagellin [30]. Those studies confirmed the importance of the conserved D1 domain for binding to the TLR5 receptor to generate a proinflammatory response.

Flagellin from different bacteria shares highly conserved regions in the extreme amino- and carboxyl-terminals of the D1 domain, which is the TLR5′s recognition and activation domain [5,27,31]. Furthermore, 13 amino acid residues described in flagellin from *S. typhimurium* have been reported as responsible for TLR5 activation in mammals, and 7 are located in the terminal amino and 5 in the terminal carboxyl of the D1 domain [5]. Clustal Omega multiple alignment showed that there is not a significant sequence homology in the amino section of the D1 domain; however, the seven amino acid residues (L88, Q89, R90, L94, Q97, N100, E114) are 100% conserved between species, including rND1. Moreover, it has been described that three amino acid residues (R89, E114, and L93) in flagellin from *B. subtilis* and *T. pallium* represent a hot spot that provides shape and chemical complementarity to bind TLR5, and the variation in these residues generates the major functional differences between flagellins with a TLR5-activator and non-activator profile [27,30], which explains why some flagellins do not bind to TLR5 and, therefore, do not generate a proinflammatory state [32]. The residues R89, E114, and L93 are present in our recombinant domain rND1 confirming its functional profile as a universal TLR5-activator. We showed that rND1 has biological activity and has no cytotoxic effects on THP-1 cells at the dose and time used in this study.

The soluble cytokines profile generated by rND1 in THP-1 cells and mononuclear cells (Mo) was consistent with the results at the transcriptional level. It is interesting that THP-1 cells were less responsive than the Mo and may be explained by a selective PAMP signaling, and we have suggested that Mo isolated from whole blood represent a more diverse cell population able to respond to TLR5, thus amplifying the cytokine production. Our results are consistent with a previous report in THP-1 cells and human peripheral blood mononuclear cells (PBMCs), where the stimulation with 10 ng/mL LPS from *P. aeruginosa* upregulated IL-6, IL-10, and IL-8 in PBMCs, with a peak at 24 h; however, THP-1 cells did not secrete IL-6 and IL-10 at any time, and IL-8 induction was substantially lower than in PBMCs [33]. rND1 significantly increased expression and secretion of Eotaxin, IL-8, and MIP1α (macrophage inflammatory protein-1 alpha), and these chemotactic cytokines are characterized by their ability to stimulate cell migration of eosinophil, basophil, macrophage, NK cell, and neutrophil to inflammatory sites [34]. Moreover, FLA and LPS showed a lower secretion profile for these cytokines, suggesting that rND1 has a greater potential as an immunomodulatory molecule than those full PAMPs; however, rND1 showed a limited ability to promote a mature profile of dendritic cells, as reported by other authors [35,36,37]. The role of flagellin in mature DC is controversial; a study performed to induce mature DCs using 1 μg/mL flagellin B from *V. vulnificus* showed no effect on CD80 and CD86 induction after 48 h of stimulation [38]. However, another report using iDC stimulated with FliC purified from *S. typhimurium* generated mature DC with increased expressions of CD83 and CD86 after 24 h [39]. On the other hand, when the D0/D1 domain from *S. typhimurium* flagellin (Flg) was fused to a cytomegalovirus (CMV) peptide-coupled αCD40 antibody (αCD40.FlgCMV), it generated the upregulation of molecules related to a mature phenotype in monocyte-derived DCs via TLR5 activation. This construct was more effective than the version without flagellin, and these effects were abolished by the introduction of the two mutations (R90A and E114A) into the flagellin domain [40].

The present study has demonstrated that rND1 induces the expression of genes involved in an early inflammatory response and a profile of secreted proteins with chemotactic activity in human immune cells in a dose- and time-dependent manner, and the magnitude of the response may probably be related to the cell types exposed to this molecule. Although our recombinant domain fails to generate a profile of mature dendritic cells, it is still a good candidate to be used in combination with other antigenic peptides or PAMP. We recognize the limitations of our experimental approach; however, the previous results obtained in teleost fish have demonstrated, in vivo, that rND1 in combination with a commercial vaccine promotes an early proinflammatory response at 4 h post-injection and a greater secondary cellular response at 72 h post-injection [26]. In addition, the preliminary results generated in our laboratory, using human PBMC challenged with rND1 alone or in combination with human tumor-derived extracts, suggest, for some tumor types, a synergistic effect on IL-1b expression using the combination, compared to the extract without rND1 (unpublished data). Therefore, we suggest that rND1 could be used as an adjuvant in future therapies based on PAMPs.

## 4. Materials and Methods

### 4.1. Samples

Mononuclear cells were freshly isolated from leukocyte concentrate (buffy coat) collected from healthy donor volunteers (n = 12) enrolled in the blood bank unit of Fundación Arturo López Pérez (FALP) during the process of an altruistic blood donation for therapeutic purposes. Prior to use, all samples were tested serologically against infectious diseases. Each blood donor was informed, signed a consent letter, and authorized the use of remaining leukocytes for research purposes. All biological material and its associated information were anonymized. This research was reviewed and approved by the Institutional Scientific and Ethical Committee of Instituto Oncológico Fundación Arturo López Pérez (Santiago, Chile) on 12 May 2020 (Identification code is 2020-008-RES-SIN-INT).

### 4.2. Expression and Purification of Recombinant D1 Domain

The recombinant rND1 domain was generated and tested as previously reported [22]. The presence of contaminating LPS was removed from the purified recombinant domain utilizing a detoxi-Gel Endotoxin removing Gel (Invitrogen, 20339), following the manufacturer’s recommendations. The residual LPS content for each aliquot was determined using Chromogenic LAL Endotoxin Quantitation Kit (Pierce, 88282), and only aliquots with endotoxins below 0.3 EU/mL were used in this research.

### 4.3. Isolation of Human Peripheral Blood Mononuclear Cells (PBMCs)

The buffy coat was diluted with phosphate buffered saline (PBS) (HyClone, SH30256) in a 1:1 ratio. A total of 30 mL of this suspension was layered over a volume of 10 mL lymphocyte separation medium (density of 1.077–1.080 g/mL, Corning, NY, USA, 25-072-CV). Gradients were subjected to centrifugation at 400× *g* for 40 min at room temperature, and PBMC were recovered from the interface. PBMC were washed with PBS, and red blood cells were lysed using BD pharm Lyse^TM^ (BD Biosciences, 555899, San Jose, CA, USA). PBMC were washed with PBS, and the viable cell concentration was determined by Trypan blue exclusion.

### 4.4. Cell Culture and Treatments

#### 4.4.1. THP-1 Cell Line

The human monocyte cell line THP-1 was obtained from European Collection of Authenticated Cell Cultures (ECACC, 88081201) and was cultured in an RPMI-1640 medium (HyClone, SH30027), supplemented with 10% fetal bovine serum (FBS) (Corning, 35-010), 100 IU/mL penicillin, and 100 mg/mL streptomycin (Corning) at 37 °C with 5% CO_2_ in 75 cm^2^ flasks. The cells were dispensed into 12-well plates at a concentration of 1 × 10^6^ cell/mL in RPMI-1640 medium, supplemented with 1% FBS, 100 IU/mL penicillin, and 100 mg/mL streptomycin (Corning) at 37 °C with 5% CO_2_ and incubated with 1 µg/mL rND1, 50 ng/mL flagellin from *S. typhimurim* (FLA) (InvivoGen, tlrl-stfla), 1 µg/mL lipopolysaccharide (LPS) from *E. coli* K12 strain (InvivoGen, tlrl-eklps), and a control of cells without treatment, respectively, by 3 h. After treatment, total RNA was extracted from cells, and the cell culture supernatant was used for determination of cytokines by Luminex.

#### 4.4.2. PBMC

Isolated PBMCs were adjusted to 6 × 10^6^ cell/mL and dispensed at a concentration of 3 × 10^7^ cell in 100 mm plates and cultivated to 37 °C with 5% CO_2_ in RPMI-1640 medium (HyClone) supplemented with 10% FBS (Corning), 100 IU/mL penicillin, and 100 mg/mL streptomycin (Corning). Mononuclear cells (Mo) in monolayer were obtained after overnight culturing. These cells were washed twice with PBS and exposed to different doses (0.01, 0.05, 0.1, 0.5, and 1 µg/mL) of rND1 in different times (5, 30, 90, 180, and 360 min), followed by total RNA extraction. Both experiments were performed in RPMI-1640 medium supplemented with 1% FBS, 100 IU/mL penicillin, and 100 mg/mL streptomycin (Corning) at 37 °C with 5% CO_2_. Totals of 50 ng/mL FLA (*S. typhimurim*, InvivoGen, Vista Sorrento Pkwy San Diego, CA, USA) and 1 µg/mL LPS (*E. coli* K12, InvivoGen) and cells without treatment were used as controls. The cell culture supernatant was used for the determination of cytokines by Luminex.

#### 4.4.3. Monocytes-Derived Dendritic Cells (MoDC)

After PBMC isolation, the CD14+ population was separated using magnetic beads (130-050-201 MACS, Miltenyi Biotec, Germany), following the manufacturer’s recommendations. The isolated CD14+ cells 8 × 10^5^/mL cells were seeded in 6-well flat-bottom plates (Falcon, USA) and cultured with 5 mL volume/well for 7 days in supplemented RPMI 1640 (HyClone) with 10% FBS (Corning), 100 IU/mL penicillin, 100 mg/mL streptomycin (Corning), 800 IU/mL GM-CSF (R&D Systems, Minneapolis, MN, USA), and 500 IU/mL IL-4 (R&D, USA) at 37 °C with 5% CO_2_. After 6 days, the differentiated dendritic cells were treated by 24 h with LPS (1 µg/mL) (*E. coli* K12, InvivoGen), rND1 (5 µg/mL), FLA (100 ng/mL) (*S. typhimurim*, InvivoGen), and medium alone (as control). Finally, all treated cells were recovered and analyzed by flow cytometry.

### 4.5. MTT Assay

To exclude cytotoxicity effects by rND1, THP-1 cells were dispensed into 96-well plates at a concentration of 5 × 10^4^ cell/well in 100 μL of RPMI-1640 (Biological Industries 01-103-1A) medium, supplemented with 1% FBS, 100 IU/mL penicillin, and 100 mg/mL streptomycin (Corning) for 6 h at 37 °C with 5% CO_2_. Then, cells were treated with 1 µg/mL of rND1, 1 µg/mL lipopolysaccharide (InvivoGen, tlrl-eklps), 10% DMSO (Winkler DI-0755), PBS (vehicle control), and a control only medium by 3, 6, 24, 48, and 96 h, respectively. After incubation with 10 μL of MTT solution (Life Technologies M6494) (5 mg/mL in PBS) for 3 h at 37 °C, 100 μL of DMSO (Winkler DI-0755) was added to each well to dissolve the formazan crystal. After 30 min at 37 °C, the absorbance was read at 570 nm using Cytation 5^®^ plate reader (BioTek, Santa Clara, CA, USA).

### 4.6. Determination of Cytokines

MILLIPLEX MAP Human Cytokine/Chemokine Magnetic Bead Panel kit (HCYTOMAG60, Merck Millipore, Darmstadt, Alemania) was used to measure the cytokines levels from cell culture supernatant in THP-1 and Mo cells stimulated with rND1, according to the manufacturer’s instructions, in a MAGPIX^®^ System (Merck, Darmstadt, Germany). All samples were run in duplicate, and basal culture medium was used as blank. The cytokines included were EGF, Eotaxin, G-CSF, GM-CSF, IFNa2, IFNy, IL-10, IL12p40, IL12p70, IL13, IL15, IL17, IL1ra, IL1α, IL1β, IL2, IL3, IL4, IL5, IL6, IL7, IL8, IP10, MCP1, MIP1α, MIP1β, TNFα, TNFβ, and VEGF. The concentration values were obtained from the mean fluorescent intensity (MFI) by using platform MAGPIX^®^ (software Milliplex Analyst v 3.5.5.0). For each target, a 5-point standard curve was generated from the reference cytokines pool.

To reduce the risk of parallel pathways with pro- and anti-inflammatory downstream effectors during in vitro studies, we analyzed by Luminex the cytokine composition of the cell culture medium supplemented with 1% FBS (Supplementary Materials, Table S4).

### 4.7. Flow Cytometry Analysis of MoDCs

On day 7, monocyte derived dendritic cells from each well were collected by gently pipetting, and cells were washed in PBS1x/FCS1% solution by centrifugation at 1400 rpm for 5 min. APC-anti-human CD11c (Becton Dickenson, 559877), FITC-anti-human CD83 (Becton Dickenson, 556910), PE-anti-human CD86 (Becton Dickenson, 55658), V450-anti-human CD40 (Becton Dickenson, 561219), and PerCP-Cy5.5-anti-human HLA-DR (Becton Dickenson, 560652) antibodies were added at a 1:25 or 1:50 dilution and incubated for 30 min on ice in the dark. The cells were washed in PBS1x/FCS1% solution and fixed in Cytofix solution (Becton Dickenson, 554655). The specific fluorescent labeling was analyzed at FACSCanto II flow cytometer (Becton Dickenson), and 10,000 events were acquired.

For analysis of the expression of TLR5 in monocytes (Mo, CD14+), THP-1 cells, and MDA-231 cells (breast cancer cell line), the cells were collected from a regular cell culture, washed in PBS1x/FCS1% solution, and centrifuged at 1400 rpm for 5 min. PE-anti-human TLR5 (# 394504) antibody (BioLegend, San Diego, CA, USA) was added at 1:50 dilution and incubated for 30 min on ice (in the dark). After washing in PBS1x/FCS1% solution, the cells were fixed in Cytofix solution (# 554,655 (BD). The specific fluorescent labeling was analyzed at FACSCanto II flow cytometer (BD), and 10,000 events were acquired for analysis.

### 4.8. Phagocytosis Assay

To measure the efficiency of phagocytosis, as a functional parameter of differentiation in immature and mature dendritic cells, measurements were performed using fluorescent dextran and flow cytometry. Dextran uptake activity was assessed by incubating 2.5 × 10^5^ MoDC (medium, LPS, rND1, FLA) with 0.12 µg/µL of FITC-conjugated dextran (Sigma, Darmstadt, Alemania, FD40S-100) for 2 h at 37 °C in the dark. Later, cells were washed twice with PBS1x/FCS1%, and the internalized dextran was quantified in a FACSCanto II flow cytometer (BD).

### 4.9. RNA Extraction and cDNA Synthesis

Total RNA was extracted from THP-1 and Mo cells using the Rneasy Mini Kit (Qiagen, Germany). The RNA concentrations and purity were measured using a spectrophotometer (Cytation 5, BioTek, Winooski, VT, USA) and stored at −80 °C.

For differential gene expression assays, 350 ng of total RNA was used for cDNA synthesis, which was performed using the PrimeScript^TM^ RT Reagent Kit with gDNA Eraser (Takara Bio, San José, CA, USA), according to the manufacturer’s instructions. A negative control to check for genomic DNA contamination was included in the sample group, for that total RNA from a sample pool was treated with DNAse without reverse transcriptase reaction.

### 4.10. Analysis of Gene Expression

Gene expression analyses were performed on THP-1 and Mo cells treated with recombinant rND1 domain using specific primers, as described in Table S5 (Supplementary Materials). The qPCR amplification efficiency for each primer pair was determined using 1:5 serial cDNA dilutions from PBMC. The amplification efficiency was calculated considering the value of the slope from each trend line; cycle threshold (CT) results were plotted as a function of the log10 values for each dilution, according to the equation E = 10 (1/slope) [41]. The specificity of the qPCR products for each pair of primers was confirmed by melting curve analysis. Amplification efficiencies resulted between 87% and 97%, which were suitable for gene expression analyses (GAPDH = 90%, β-actin =90%, IL-8 = 87%, TNF-α =N92%, IL-1β =97%).

Real-time PCR analyses were performed at the QuantStudio 3 thermocycler (Applied Biosystems, Waltham, MA, USA) using the Brilliant II SYBR Green qPCR Master Mix kit (Agilent Technologies, Santa Clara, CA, USA). Each amplification reaction was performed in a final volume of 12 μL, consisting of 6 μL of buffer, 500 nM primers, 300 nM ROX (50 nM), and 2 μL of cDNA diluted 1:10. The PCR program consisted of a 10 min activation and denaturation step at 95 °C, followed by 40 cycles of 15 s at 95 °C, 30 s at the annealing temperature of the corresponding primers, and an additional 15 s extension at 72 °C. The melting curve was analyzed to examine the specificity of the reaction in each well and to verify the absence of primer dimers and non-specific amplification products.

Three biological replicates were used, and each qPCR was performed in duplicate. The reactions included a negative control without reverse transcriptase to check for genomic DNA contamination and a negative control without a template to check for the presence of primer dimers. All genes were quantified from the same batch of cDNA. Ct values, obtained using QuantStudio^TM^ Design and Analysis Software v1.5.1 (Applied Biosystems, Waltham, MA, USA), were transformed into relative expression units using the comparative Ct method (2^−ΔΔCt^), where Ct was a cycle threshold [42]. GAPDH and β-actin (actin beta) were selected as housekeeping genes for normalization. Fold change was defined based on a control collected in parallel with each experiment.

### 4.11. Statistical Analysis

Statistical analysis was performed using GraphPad Prism software, version 9.0.1. Differences in mean values between groups of cells treated with the recombinant domain and the untreated control were analyzed by unpaired student t-test and nonparametric Mann–Whitney test. One-way ANOVA and Tukey’s multiple comparison test were performed for statistical analysis between various variables. The critical value for statistical significance was established as *p* ≤ 0.05. Values marked with asterisks mean the following: * *p* < 0.05; ** *p* < 0.01, *** *p* < 0.001, and **** *p* < 0.0001.

## Data Availability

The authors confirm that the data supporting the findings of this study are available within the article and/or its Supplementary Materials.

## Supplementary Materials

The following supporting information can be downloaded at: https://www.mdpi.com/article/10.3390/molecules28052394/s1, Figure S1: Flow cytometry analysis for CD83, Figure S2: Expression of costimulatory and HLA-DR molecules in MoDCs treated with rND1, Table S1: Cytokines expression between blood donors, Table S2: Mean and confidence intervals (95% CI) of soluble proteins secreted by THP-1 cells after of stimulation with rND1, Table S3: Mean and confidence intervals (95% CI) of soluble proteins secreted by Mo cells after of stimulation with rND1, Table S4: Soluble protein in the cell culture medium supplement with 1% FBS, Table S5: Primer and sequences used for gene expression analysis by quantitative real time PCR.
