# Supplementary material for: Recombinant Domain of Flagellin Promotes In Vitro a Chemotactic Inflammatory Profile in Human Immune Cells Independently of a Dendritic Cell Phenotype
Source: Molecules. 2023 Mar 5;28(5):2394. doi: 10.3390/molecules28052394 (PMC10005431; doi:10.3390/molecules28052394)
Supplement: Supplementary file 1 [file molecules-28-02394-s001.zip › molecules-2210022-supplementary.pdf]

Supplementary Materials

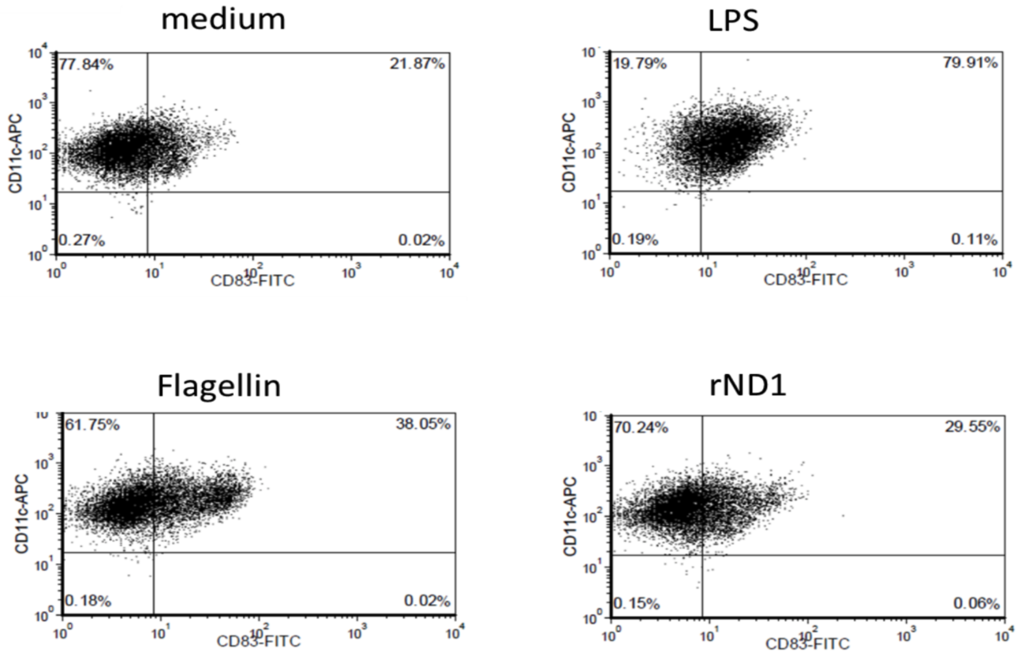

Figure S1. Flow cytometry analysis for CD83. Dot plot shows the distribution and percentage of cells expressing CD83 within the gate CD11c positive for iDC (only medium), mDC (LPS), and MoDC treated with FLA, and rND1. The dot plot show a representative experiment. LPS: lipopolysaccharide from *E. c*oli K12 strain (1 μg/mL), FLA: Flagellin from *S. typhimurim* (100 ng/mL), rND1: recombinant amino terminus of the D1 domain of *V. anguillarum* flagellin (5 μg/mL).

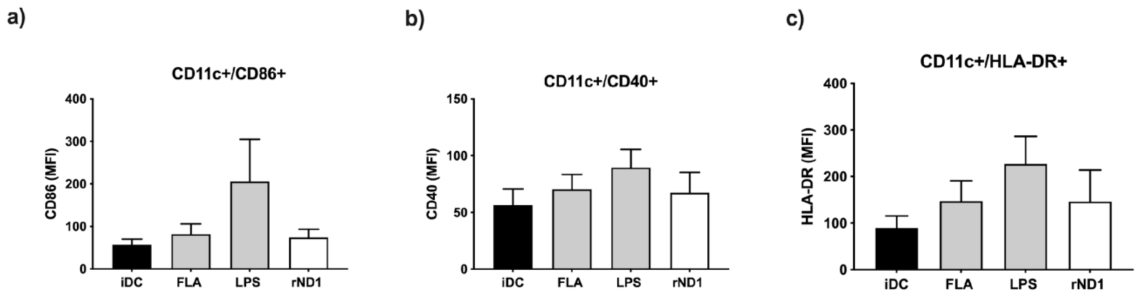

Figure S2. Expression of costimulatory and HLA-DR molecules in MoDCs treated with rND1. The bar graphs show the median fluorescence intensity of CD86 (a), CD40 (b) and HLA-DR (c) on the surface of CD11c positive cells after treatment with FLA and rND1. As controls MoDCs were cultured in presence of LPS for mDC and only culture medium for iDC. The values represent the means of three independent experiments. iDC: immadure dendritic cell; LPS: lipopolysaccharide (1 μg/mL), FLA: Flagellin from *S. typhimurim* (100 ng/mL), rND1: recombinant amino terminus of the D1 domain of *V. anguillarum* flagellin (5 μg/mL).

Table S1. Cytokines expression between blood donors

Treatment 3h			rND1 1 μg/ml Fold change		
Gender	ABO	Rh	IL-8	IL-1β	TNF-α
*Female ●	A	+	7.1	15.5	7.1
Female ●	O	+	15.9	338.5	16.1
Male ●	A	+	13.4	67.1	18.1
Male ●	O	+	5.9	29.8	12.4
Male ●	O	-	7.3	18.6	12.2
Male ●	O	+	27.8	225.1	59.9
Male ●	B	-	17.1	99.9	121.6

* rND1 0.5 ug/ml

Table S2. Mean and confidence intervals (95% CI) of soluble proteins secreted by THP-1 cells after stimulation with rND1.

	**Control**		**FLA (50 ng/ml)**		**LPS (1 μg/ml)**		**rND1 (1 μg/ml)**		**Significance**
Soluble Protein	mean (pg/ml)	95%CI	mean (pg/ml)	95%CI	mean (pg/ml)	95%CI	mean (pg/ml)	95%CI	*P-value*
**EGF**	nd	und	31.25	1.94	8.80	3.30	nd	und	ns
**Eotaxin**	nd	und	15.96	6.40	3.56	1.32	nd	und	ns
**G-CSF**	nd	und	11.72	11.48	nd	und	nd	und	ns
**GM-CSF**	0.18	0.06	2.18	0.75	0.68	0.34	0.14	0.07	ns
**IFNα2**	nd	und	6.04	3.86	1.45	2.83	nd	und	ns
**IFNy**	nd	und	1.30	1.08	0.03	0.06	nd	und	ns
**IL-10**	0.003	0.004	2.77	1.86	0.35	0.39	0.003	0.005	ns
**IL-12p40**	nd	und	3.66	3.00	0.002	0.003	nd	und	ns

**IL-12p70**	nd	und	0.40	0.32	nd	und	nd	und	ns
**IL-13**	nd	und	1.13	0.36	0.09	0.12	nd	und	ns
**IL-15**	0.01	0.01	0.94	0.11	0.17	0.11	0.01	0.01	ns
**IL-17**	0.04	0.04	1.22	0.59	0.09	0.17	0.01	0.01	ns
**IL-1ra**	15.50	8.58	79.76	3.09	56.63	34.00	11.74	1.70	ns
**IL-1α**	0.15	0.02	1.64	0.94	0.57	0.46	0.12	0.04	ns
**IL-1β**	0.22	0.16	149.84	94.08	49.91	16.64	1.11	1.72	ns
**IL-2**	0.08	0.03	0.69	0.23	0.16	0.09	0.07	0.02	ns
**IL-3**	0.01	0.01	0.02	0.02	0.01	0.01	0.002	0.002	ns
**IL-4**	0.18	0.28	7.33	4.49	1.62	0.67	0.05	0.05	ns
**IL-5**	0.02	0.01	0.23	0.13	0.05	0.03	0.02	und	ns
**IL-6**	0.06	0.01	1.55	2.13	0.22	0.17	0.08	0.07	ns
**IL-7**	0.04	0.06	4.43	1.36	1.36	1.03	0.003	0.01	ns
**IL-8**	7.65	9.39	741.00	415.42	122.69	94.18	24.05	35.69	ns
**IP-10**	0.52	0.83	215.12	155.80	167.87	126.70	1.03	1.08	ns
**MCP1**	20.36	28.09	151.00	22.09	87.31	26.26	25.42	42.70	ns
**MIP-1α**	4.49	22.10	739.00	543.68	187.00	67.41	14.06	19.04	ns
**MIP-1β**	27.46	38.10	31107.97	8787.47	2061.33	1468.80	62.26	87.16	ns
**TNF-α**	12.73	6.60	4638.00	1444.08	808.33	481.76	21.94	8.44	ns
**TNF-β**	0.19	0.09	1.40	0.45	0.36	0.23	0.18	0.07	ns
**VEGF**	5.90	6.61	41.70	7.10	19.59	6.68	5.19	7.34	ns

nd: not detectable, und: Undeterminated, ns: not signiticant

Table S3. Mean and confidence intervals (95% CI) of soluble proteins secreted by Mo cells after stimulation with rND1.

	**Control**		**FLA (50 ng/ml)**		**LPS (1 μg/ml)**		**rND1 (1 μg/ml)**		**Significance**
Soluble Protein	mean (pg/ml)	95%CI	mean (pg/ml)	95%CI	mean (pg/ml)	95%CI	mean (pg/ml)	95%CI	*p-value*
**EGF**	nd	und	2.51	2.83	7.39	14.48	22.39	20.58	ns
**Eotaxin**	1.16	1.49	8.76	4.32	12.72	11.69	17.27	10.73	0,038
**G-CSF**	nd	und	nd	und	12.91	25.31	10.15	19.90	ns
**GM-CSF**	0.53	0.58	1.68	1.39	1.39	1.94	3.27	2.93	ns
**IFNα2**	nd	und	0.01	0.02	1.61	3.15	1.75	1.99	ns
**IFNy**	0.30	0.46	0.95	0.62	2.00	3.93	2.57	3.03	ns
**IL-10**	0.38	0.48	4.86	4.41	12.11	15.36	25.76	27.30	ns
**IL-12p40**	nd	und	3.41	4.33	1.89	2.04	7.62	8.68	ns
**IL-12p70**	nd	und	0.09	0.17	1.70	3.34	0.55	0.63	ns
**IL-13**	0.08	0.11	0.28	0.22	0.17	0.33	0.86	0.97	ns
**IL-15**	0.29	0.25	0.64	0.37	0.41	0.66	1.13	1.26	ns
**IL-17**	0.47	0.36	1.03	0.62	0.52	0.47	1.22	1.04	ns
**IL-1ra**	7.37	14.29	12.47	16.57	12.72	21.31	21.61	26.61	ns
**IL-1α**	0.62	0.48	1.26	0.97	0.92	1.05	2.02	1.90	ns
**IL-1β**	0.48	0.44	3.98	1.41	6.53	4.59	13.69	11.50	ns
**IL-2**	1.37	2.29	1.99	3.02	0.48	0.08	2.88	3.33	ns
**IL-3**	0.02	0.02	0.34	0.60	0.01	0.03	0.90	1.70	ns
**IL-4**	1.14	1.23	1.83	1.43	5.15	9.81	4.45	5.01	ns

**IL-5**	0.05	0.04	0.19	0.15	0.13	0.13	0.27	0.27	ns
**IL-6**	0.20	0.12	21.50	14.45	42.40	11.17	89.91	78.04	ns
**IL-7**	0.03	0.06	0.14	0.17	1.33	2.60	2.26	3.20	ns
**IL-8**	87.91	78.55	662.25	274.41	747.00	444.91	1188.66	927.72	0,037
**IP-10**	337.75	209.93	350.25	162.45	153.00	7.84	445.25	347.01	ns
**MCP1**	89.80	128.29	291.76	447.82	40.49	5.69	529.78	898.21	ns
**MIP-1α**	9.79	2.85	136.37	85.33	301.00	223.44	2063.00	3243.19	0,046
**MIP-1β**	62.95	29.28	417.50	237.46	967.50	1082.88	9493.25	16091.40	ns
**TNF-α**	9.56	7.45	245.04	181.61	673.50	600.73	11030.00	19190.34	ns
**TNF-β**	0.51	0.36	0.80	0.30	1.02	1.28	1.37	1.28	ns
**VEGF**	0.52	0.76	3.70	4.15	8.70	17.05	23.45	22.39	ns

nd: not detectable, und: Undeterminated, ns: not signiticant

Table S4: Soluble protein in the cell culture medium supplemented with 1% FBS

Soluble Protein	pg/ml				
**EGF**	2.4408	Chi=1.68%	CV=0.29%	R2=1.00	DC=(2.66, 11891.81)
**Eotaxin**	1.3786	Chi=0.095%	CV=0.019%	R2=1.00	DC=(0.57, 741999.00)
**G-CSF**	1.2419	Chi=2.44%	CV=0.39%	R2=1.00	DC=(1.72, 9921.64)
**GM-CSF**	0.2907	Chi=7.41%	CV=0.78%	R2=1.00	DC=(0.94, 7776.03)
**IFNα2**	0.5228	Chi=1.50%	CV=0.34%	R2=1.00	DC=(0.94, 16235.99)
**IFNy**	0.8716	Chi=0.99%	CV=0.14%	R2=1.00	DC=(1.18, 10511.18)
**IL-10**	1.2473	Chi=3.11%	CV=0.50%	R2=1.00	DC=(1.94, 9797.91)
**IL-12p40**	0.6832	Chi=0.13%	CV=0.021%	R2=1.00	DC=(0.18, 387857.07)
**IL-12p70**	1.0698	Chi=5.09%	CV=0.67%	R2=1.00	DC=(1.72, 8462.08)
**IL-13**	0.8249	Chi=1.10%	CV=0.19%	R2=1.00	DC=(1.06, 22040.55)
**IL-15**	0.6199	Chi=1.60%	CV=0.23%	R2=1.00	DC=(0.99, 9732.67)
**IL-17**	0.6639	Chi=3.59%	CV=0.44%	R2=1.00	DC=(0.78, 8385.02)
**IL-1ra**	1.82	Chi=1.04%	CV=0.17%	R2=1.00	DC=(1.82, 10658.21)
**IL-1α**	0.589	Chi=1.79%	CV=0.25%	R2=1.00	DC=(1.15, 15603.51)
**IL-1β**	0.0821	Chi=11.25%	CV=2.15%	R2=1.00	DC=(0.54, 8578.90)
**IL-2**	0.1237	Chi=6.36%	CV=0.81%	R2=1.00	DC=(0.63, 8619.87)
**IL-3**	0	Chi=13.99%	CV=1.19%	R2=1.00	DC=(0.42, 7526.05)
**IL-4**	1.5518	Chi=11.83%	CV=2.04%	R2=1.00	DC=(2.19, 10238.53)
**IL-5**	0.1522	Chi=7.29%	CV=0.80%	R2=1.00	DC=(0.68, 7821.41)
**IL-6**	0.2718	Chi=9.79%	CV=1.47%	R2=1.00	DC=(0.80, 9239.01)
**IL-7**	1.3614	Chi=0.88%	CV=0.13%	R2=1.00	DC=(1.95, 19072.54)
**IL-8**	0.0178	Chi=8.69%	CV=2.15%	R2=1.00	DC=(0.25, 8285.78)
**IP-10**	1.1995	Chi=2.90%	CV=0.59%	R2=1.00	DC=(2.03, 11490.07)
**MCP1**	1.8974	Chi=1.19%	CV=0.26%	R2=1.00	DC=(2.32, 11567.25)
**MIP-1α**	1.6407	Chi=25.10%	CV=1.74%	R2=1.00	DC=(2.17, 7107.40)
**MIP-1β**	0	Chi=13.45%	CV=2.39%	R2=1.00	DC=(0.40, 7714.80)
**TNF-α**	0.3826	Chi=3.23%	CV=0.44%	R2=1.00	DC=(1.09, 8588.99)
**TNF-β**	0.3034	Chi=8.75%	CV=2.26%	R2=1.00	DC=(0.98, 8303.73)

**VEGF**	3.1891	Chi=2.98%	CV=0.70%	R2=1.00	DC=(2.94, 10043.08)

Table S5. Primer and sequences used for gene expression analysis by quantitative real time PCR.

**Gene**	**name**	**Sequence 5′-3′**	**Accesion no.**
IL-8	Interleukin 8	F AGACAGCAGAGCACACAAGC<br>R ATGGTTCCTTCCGGTGGT	AF385628.2
IL-1β	Interleukin 1 β	F GTGGCAATGAGGATGACTTGTTC<br>R TAGTGGTGGTCGGAGATTCGTA	NM_000576.3
TNF-α	Tumor necrosis factor α	F TCTTCTCGAACCCCGAGTGA<br>R CCTCTGATGGCACCACCAG	NM_000594.4
GAPDH	Glyceraldehyde-3-phosphate dehydrogenase	F AAGGTGAAGGTCGGAGTCAA<br>R AATGAAGGGGTCATTGATGG	NM_002046.7
B-actin	Actin β	F GCACAGAGCCTCGCCTT<br>R GTTGTCGACGACGAGCG	NM_001101.5
